# Patient outcomes associated with subcutaneous C1INH prophylaxis for hereditary angioedema: a retrospective analysis

**DOI:** 10.1186/s13223-023-00861-9

**Published:** 2023-12-11

**Authors:** William Lumry, Timothy Craig, John Anderson, Marc Riedl, Henry Li, Raffi Tachdjian, Michael Manning, Paolo Bajcic, Frank Rodino, Sam Wang, Thomas R. Sexton, Jonathan A. Bernstein

**Affiliations:** 1 Allergy & Asthma Specialists of Dallas, 10100 N. Central Expressway, Suite 100, Dallas, TX 75231 USA; 2 Allergy Asthma and Immunology, Departments of Medicine, Pediatrics, and Biomedical Sciences, Penn State University, Hershey, PA USA; 3 AllerVie Health, Birmingham, AL USA; 4 Division of Rheumatology, Allergy and Immunology, University of CA—San Diego, La Jolla, CA USA; 5 Institute for Asthma & Allergy, Chevy Chase, MD USA; 6 Pediatric Allergy and Immunology, UCLA School of Medicine, Los Angeles, CA USA; 7 Allergy, Asthma & Immunology Associates, Ltd, Scottsdale, AZ USA; 8 CSL Behring, King of Prussia, PA USA; 9 Churchill Outcomes Research, LLC, Red Bank, NJ USA; 10 ICON Plc, Gaithersburg, MD USA; 11 Stony Brook University, Stony Brook, NY USA; 12 Division of Rheumatology, Allergy and Immunology, University of Cincinnati, Cincinnati, OH USA; 13 Bernstein Allergy Group, Inc., Cincinnati, OH USA

**Keywords:** C1-inhibitor, Hereditary angioedema, Prophylaxis, Qualitative research, Quality of life, Real-world, Retrospective chart review, Subcutaneous C1INH

## Abstract

**Background:**

Real-world data on subcutaneous C1INH (C1INH[SC]) usage and patient-level impacts on hereditary angioedema (HAE)-related outcomes and quality of life (QoL) are both lacking and challenging to generate using conventional study methodologies. Using a hybrid study design involving patient interviews supplemented by retrospective medical chart data review, we conducted a real-world assessment of the impact of C1INH(SC) prophylaxis on HAE attack patterns, QoL, and on-demand medication use.

**Methods:**

The study was conducted at seven US sites and included 36 adults with HAE who had been treated with C1INH(SC) long-term prophylaxis following ≥ 12 months of on-demand management only. Patients underwent 30-min interviews, facilitated and analyzed by a trained qualitative research specialist. Medical records were reviewed for 12 months before (pre-index) and after (post-index) initiation of C1INH(SC). Using interview data with descriptive terms converted to numerical values, we compared pre- versus post-index attack frequency, severity, and rescue medication usage.

**Results:**

Mean (SD) annualized attack frequency per patient decreased 82.0%, from 38.8 (38.8) attacks/year pre-index to 7.0 (15.3) attacks/year (P < 0.001); the median number of attacks decreased by 97.0% (30 pre-index to 1 post-index). For 20 patients, the annualized attack rate after starting C1INH(SC) prophylaxis was ≤ 1 attack/year; 12 of these patients reported 0 attacks. Mean (SD) attack severity (scale: 0 = none/mild to 4 = very severe) decreased from 2.3 (0.7) pre-index to 0.9 (0.9) post-index (P < 0.001). Mean/median rescue medication use decreased by 77.2%/96.3%. Improved QoL was narratively described for many domains.

**Conclusions:**

These real-world findings indicate that long-term prophylaxis with C1INH(SC) markedly improves important factors that contribute to the goal of achieving total disease control and normalization of patients’ lives, including fewer and less severe attacks, less rescue medication usage, and improved QoL.

## Background

Hereditary angioedema (HAE) is a rare but highly burdensome disease characterized by painful, recurrent attacks of edema that are debilitating, potentially life-threatening, and detrimental to multiple facets of quality of life (QoL) [1–7]. The stated consensus goal of HAE management is to “achieve total control of the disease and to normalize patients’ lives,” which can “only be achieved by long-term prophylactic treatment” [8]. Recent decades have witnessed the emergence of a number of disease-specific therapies for long-term prophylaxis (LTP), making this goal increasingly achievable for many patients.

Subcutaneous (SC) plasma-derived C1INH (C1INH[SC]; HAEGARDA^®^, CSL Behring) is one of the recommended first-line options for LTP in current HAE management guidelines [8, 9]. Prospective clinical trial data with C1INH(SC) demonstrated significant reductions in HAE attack frequency and on-demand rescue medication use, along with improved health-related quality of life (HRQoL) outcomes compared with placebo [10, 11]. Despite FDA approval in 2017, there remains a lack of real-world data on C1INH(SC) usage and patient-level impacts on attack frequency and QoL. In fact, the gathering of such data can be challenging, given that HAE patient information is often widely dispersed among a variety of clinical settings. In addition, manufacturers of HAE medications often distribute HAE products through specialty pharmacies while maintaining proprietary data hubs. Finally, most current prophylactic and on-demand therapies can be self-administered, so formal documentation of attacks managed outside of the clinic is far less likely than in the past.

Given these complexities, triangulating information from multiple data sources is needed to provide a broader and more accurate assessment of treatment outcomes.

We used a hybrid study design combining semi-structured, qualitative patient interviews supplemented by traditional medical records data collection. The objective of this study was to obtain a real-world assessment of the impact of C1INH(SC) prophylaxis on HAE attack frequency and severity, as well as related effects on QoL and on-demand rescue medication use.

## Methods

### Study design and patients

This study was a hybrid design combining semi-structured, qualitative interviews used in parallel with a retrospective medical records review. Participants were identified from the practice populations of seven clinician-investigators in the United States who were all highly experienced in the treatment of patients with HAE. The study was conducted in accordance with all ethical principles having their origin in the Declaration of Helsinki. All participants provided written, informed consent for the interview process. The study did not entail any prospective methodology involving patient treatment. All data were de-identified. The study protocol and documents were approved by a central institutional review board (IRB) (Advarra) and local IRBs, as required.

Participants were adults (aged ≥ 18 years) with HAE type 1 or 2 who had been using C1INH(SC) as LTP for ≥ 1 year and who were managing their HAE through on-demand therapy only for ≥ 12 months before starting C1INH(SC). Major exclusion criteria included a diagnosis of HAE with normal C1INH, concomitant autoimmune disease, concurrent use of omalizumab for urticaria, and current enrollment in a clinical trial or interventional study. Each participant agreed to a one-on-one interview and granted permission for data relevant to the study to be extracted from their medical records.

### Data collection

At each study site, the investigator designated a staff member to serve as the site coordinator, review medical chart information, and enter the required data into the study’s electronic data capture system (Medrio). Medical records were reviewed to encompass the period 12 months prior to being prescribed C1INH(SC) (pre-index), the visit at which C1INH(SC) was prescribed (index visit), and the 12-month period following the start of C1INH(SC) prophylaxis (post-index period). Data collected from charts included patient demographics, HAE type, age at initial diagnosis, family history, HAE attack rates pre- and post-index, C1INH(SC) dosing prescribed at the index visit, means of administration, and on-demand attack treatments used.

Subject interviews were conducted by telephone. Each interview was 30 min in length and conducted by one of two trained interviewers from a global clinical research organization (ICON plc) using a semi-structured interview format with open-ended questions (interview guide can be found in the online appendix). Each patient received a $75 gift card as compensation for participation.

### Data analysis

Interviews were audio-recorded, transcribed, and thematically analyzed by experienced qualitative research specialists from ICON plc. Analysis was performed using qualitative methods (MAXQDA software, Berlin, Germany) to identify themes and information relating to HAE attack frequency and severity, treatment of attacks, and impact of HAE on the patient’s life.

#### Attack frequency and severity

Attack frequency data were gathered from both medical charts and subject interviews. Attack frequency, as described verbally by each subject, was converted as necessary into a numerical annualized number of attacks.

During interviews, patients were asked about the severity of HAE attacks during the pre-index and post-index periods. For the purpose of statistically comparing mean attack severity between pre-index and post-index periods, verbal severity descriptions were translated into numerical severity scores based on a scale of 0 = none/very mild, 1 = mild, 2 = moderate, 3 = severe, and 4 = very severe. Responses that included a combination of severities were scored at the middle point between the two numerical scores for the stated severities (e.g., “mild to moderate” was given a severity score of 1.5; “moderate to severe” was given a severity score of 2.5).

#### Rescue medication usage

From patient interview transcriptions, patients’ verbal descriptions of on-demand medication usage frequency (e.g., “Did not use,” “Used for every attack,” “Used for about 25% of attacks”) were translated into an approximated numerical percentage. This percentage was multiplied by the patient’s annual attack frequency to estimate the on-demand medication usage during pre- and post-index periods for each patient individually.

#### Statistical methods

Pre- and post-index values of attack frequency, attack severity, and rescue medication use were tested for statistical significance at the 5% level using matched-pairs t-tests. Analyses were performed for all patients who had relevant data for both pre- and post-index periods. For statistical rigor, separate analyses were planned based on interview data and based on chart data, and limited to subjects who had both pre- and post-index outcome data from each source. Statistical analyses were performed using Statistix v10.0 software (Analytical Software, Tallahassee, FL).

## Results

### Study cohort

A total of 39 eligible subjects were invited to participate, three of whom did not respond to the request (one subject each from three different sites). Thus, the study included 36 subjects from seven study centers ranging in age from 24 to 77 years (mean age, 47.9 years) (Table 1). Almost two-thirds of the patients were female (64%), and a majority (89%) were White. All but two patients had HAE type 1. A family history of HAE was reported for 81% of patients. Dates of C1INH(SC) initiation (index date) ranged from January 2015 to August 2020. Subject interviews were conducted between March 2022 and January 2023.

**Table 1:** Sociodemographic and clinical characteristics of study cohort

Characteristic	Distribution (N = 36)
Age at interview (years), mean (SD), range	47.9 (15.6), 24–77
Female, n (%)	23 (64%)
Race/ethnicity, n (%)	
White	32 (89%)
Black or African American	3 (8%)
Other^a^	1 (3%)
Weight^a^ (lbs), mean, range	199.3, 123–375 (n = 28)
HAE type, n (%)	
Type 1	34 (94%)
Type 2	2 (6%)
Age at initial HAE diagnosis (y), mean, range	17.1, 4–44 (n = 32)
Family history of HAE, n (%)	29 (81%) (n = 34)
Working status	
Working full-time	22 (61%)
Retired	6 (17%)
Working part-time	2 (6%)
Self-employed	2 (6%)
Permanently unable to work / disability	2 (6%)
Looking after home/family or unemployed/seeking work	2 (6%)
Insurance type^b^	
Private health insurance	25 (69%)
Medicare/Medicaid	11 (31%)
Other	5 (17%)

^a^“Other” reported as multiracial (White/Native Hawaiian/Hispanic/Latino)

^b^Some patients reported multiple forms of insurance

N = 36 unless otherwise noted. Categorical percentages may not add up to 100 due to rounding

### C1INH(SC) dosing and administration

As recorded in medical charts at the index or first post-index visit, the prescribed dose of C1INH(SC) was 60 IU/kg in 27 (75%) patients, 40 IU/kg in 5 (14%) patients, and “other” in 4 (11%) patients (50, 50, 30, and 21 IU/kg).

All patients were prescribed C1INH(SC) at a frequency of approximately twice weekly, recorded specifically as biweekly (n = 24) or every 3–4 days (n = 11) (not recorded for 1 patient). Based on interview data, nine patients described having changed the dose and/or frequency of administration of C1INH(SC). Two patients changed their dosage or frequency secondary to having gained weight during pregnancy. Three patients reported using “trial and error” to find the frequency of treatment that they felt worked best for them. One patient tried every 3–4 days, every 5 days, and every 6 days, finally discovering that every 5 days worked best. Two other patients tried dosing every 4 days or twice weekly, but settled on dosing every 3 days. Three patients reported a change in C1INH(SC) dose or frequency of administration because of a change in body weight. One patient reported concomitant intravenous therapy (unspecified indication) and occasional local irritant reactions to the catheter (described as a “histamine” reaction) that would sometimes trigger an HAE attack; an increase in the prophylactic dose of C1INH(SC) prevented further reactions of this type.

### Annualized attack frequency and severity

Among all 36 subjects, interview-sourced data regarding both pre- and post-index attack frequency were available for 35 (97.2%) subjects; one subject did not describe pre-index attack frequency during the interview. From medical charts, both pre- and post-index attack frequency data were available for 20 out of 36 (55.6%) subjects. Given the difference in data completeness between the two source types, the interview data emerged as the dominant focus of the analysis.

**Fig. 1:**
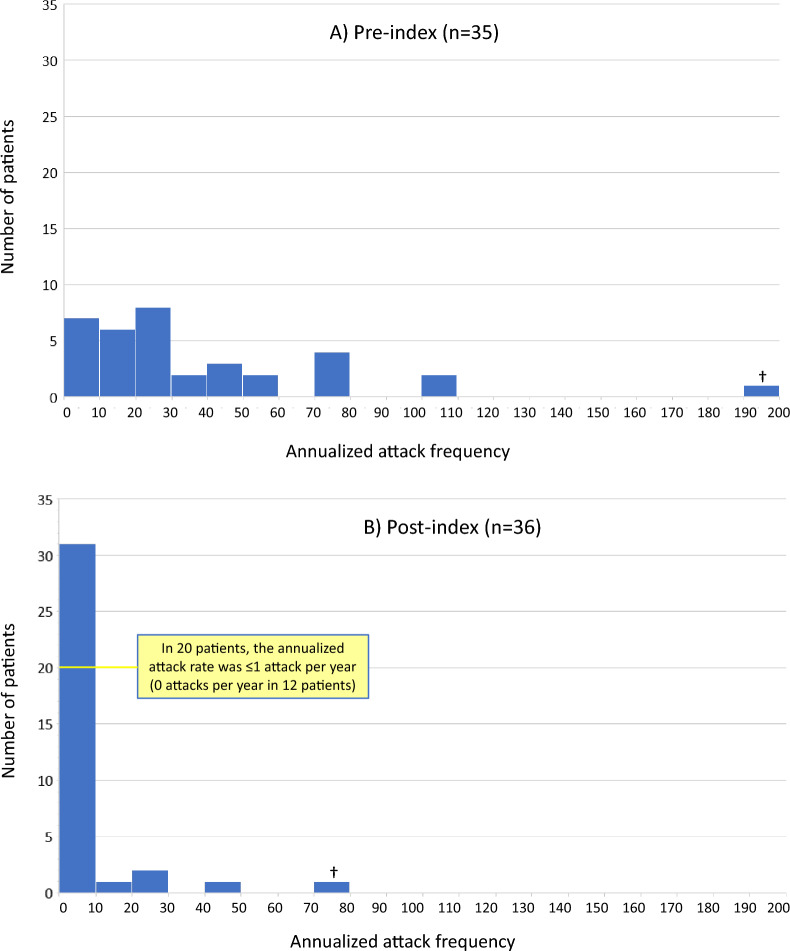
Patient distribution of annualized HAE attack frequency, based on interview data. Index = initiation of long-term prophylaxis with C1INH(SC). ^†^One outlier patient with particularly burdensome disease reported experiencing a pre-index annualized frequency of ~ 198 attacks per year (15–18 attacks per month). Post-index, the annualized rate decreased to 78 attacks per year (6–7 per month). *C1INH(SC)* subcutaneous C1-inhibitor, *HAE* hereditary angioedema

Based on interview data, the pre-index annualized attack frequency ranged from 1 to 198 attacks per year (n = 35). The post-index annualized attack frequency ranged from 0 to 78 attacks per year (n = 36). The histogram in Fig. 1 presents the categorical distribution of patients according to reported number of annual HAE attacks before and after starting C1INH(SC) prophylaxis (e.g., 0–10, 10–20, 20–30 attacks per year). Pre-index (Fig. 1A), the histogram profile was relatively flat, with patients distributed broadly over categories ranging from 0 to 100 attacks per year. Post-index (Fig. 1B), the histogram profile was skewed markedly toward the lowest categories, with the great majority of patients (n = 31; 86%) falling into the 0–10 annual attacks category. Most notably, for 20 patients, the annualized attack rate after starting C1INH(SC) was ≤ 1 attack per year, and 12 of these patients reported 0 attacks.

**Fig. 2:**
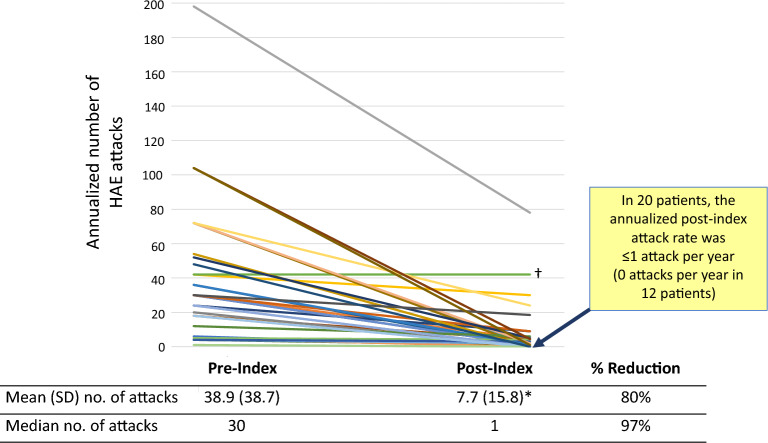
Annualized HAE attack frequencies by individual patients, pre- and post-index (n = 35), based on patient interviews. Index = initiation of long-term prophylaxis with C1INH(SC). Data for 35 patients are represented; some lines represent overlapping data points for individual patients. ^† ^This patient reported ~ 42 severe attacks per year pre-index. Frequency and severity were not notably different post-index, but the patient reported that the attacks resolved more quickly while using C1INH(SC) prophylaxis. It should also be noted that this patient’s medical chart noted a post-index attack frequency of one attack per month. *P < 0.001, pre-index versus post-index. *C1INH(SC)* subcutaneous C1-inhibitor, *HAE* hereditary angioedema, *SD* standard deviation

Individual patient attack rates as reported for pre- and post-index periods are illustrated in Fig. 2. Based on data from the 35 subjects with both pre- and post-index attack frequency information from interviews, the mean (SD) attack frequency decreased significantly from 38.9 (38.7) attacks per year pre-index to 7.7 (15.8) attacks per year post-index (P < 0.001). This represented an 80.2% decrease in the number of attacks, or a mean of 30.9 fewer attacks per patient per year. The median (interquartile range) number of annualized attacks decreased from 30 (18–52) pre-index to 1 (0–5.4) post-index, representing a 97% decrease in the median number of attacks per year.

One outlier patient with particularly burdensome disease reported experiencing an annualized frequency of approximately 198 attacks per year (15–18 attacks per month) pre-index. Post-index, the annualized rate decreased markedly, for this patient, to 78 attacks per year (6–7 per month), which represented a 60.6% decrease in yearly attacks. Further, the severity of this patient’s attacks changed from moderate to severe during the pre-index period to mild during the post-index period. In one other patient, the reported number of annualized attacks was not different between the pre- and post-index periods at approximately 42 severe attacks per year. This patient reported that, although attack severity remained severe post-index, the attacks resolved more quickly while using C1INH(SC).

**Fig. 3:**
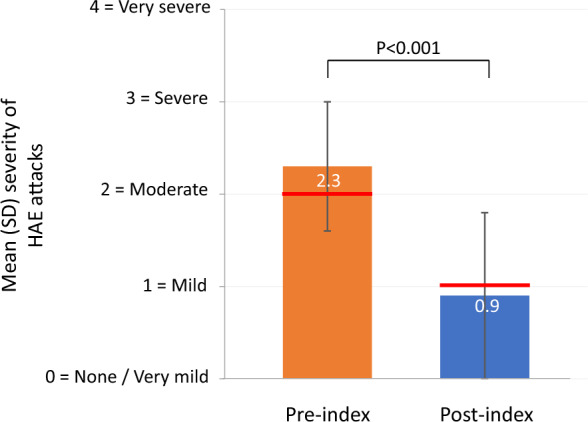
Mean reported severity of HAE attacks extrapolated from patient interviews (n = 35), pre- and post-index. Index = initiation of long-term prophylaxis with C1INH(SC). Red lines represent median values. *C1INH(SC)* subcutaneous C1-inhibitor, *HAE* hereditary angioedema, *SD* standard deviation

Based on interview data, the mean (SD) severity of attacks decreased significantly from 2.3 (0.7) during the pre-index period to 0.9 (0.9) during the post-index period (P < 0.001), on a scale ranging from 0 = none/mild to 4 = very severe (Fig. 3). One patient, who was pregnant at the time of the interview, reported no attacks during the pregnancy until 2 weeks before the interview, when she experienced what she felt to be either one prolonged attack that continued to flare up in between three separate administrations of recombinant C1INH over a period of about 10 days or a series of three individual attacks during that period. The attack activity was described to be on par with the worst attacks she had ever experienced.

A supporting analysis was performed on data from the subgroup of 20 subjects with both pre- and post-index attack frequency information in their medical charts. Similar to the interview data findings, mean (SD) attack frequency demonstrated a significant decrease from 35.0 (39.7) attacks per year pre-index to 4.7 (8.6) attacks per year post-index (P < 0.01), or a mean of 30.4 fewer attacks annually (86.6% decrease). Attack severity data could not be meaningfully analyzed based on chart data, as relevant information was obtainable from the medical charts of only 9 subjects.

### Rescue (on-demand) medications

Pre- and post-index rescue medication usage rates could be estimated based on interview content for 34 patients.

Mean annualized rescue medication use decreased by 77.2% from an estimated mean (SD) 25.4 (37.5) uses per patient per year (PPPY) pre-index to an estimated 5.4 (13.7) uses PPPY during the post-index period (P < 0.001).

Median (IQR) values for estimated pre- and post-index annualized rescue medication use, respectively, were 14.7 (3.5–30)and 0.55 (0–5) (96.3% decrease).

### Quality of life

**Fig. 4:**
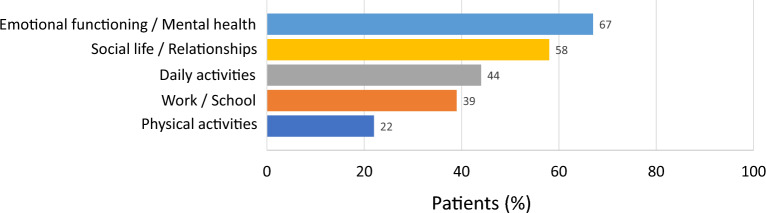
Patient-reported subjective improvements related to quality-of-life domains while using C1INH(SC). Data represent the proportion of patients whose interview content included mention of improved aspects of life categorized within one of the five QoL domains. Transcribed interviews were thematically analyzed by experienced qualitative research specialists from ICON plc

Analysis of interview data revealed a high burden of QoL impairment related to HAE, with 75% to 89% of patients indicating negative impacts of their disease on various QoL domains including emotional functioning/mental health (89% of patients), social life/relationships (86%), daily activities (75%), work/school (83%), and physical activities (75%). While using C1INH(SC), substantial proportions of patients also indicated experiencing improvement in many of these domains, including emotional functioning/mental health (67% of patients), social life/relationships (58%), daily activities (44%), work/school (39%), and physical activities (22%) (Fig. 4).

## Discussion

HAE management is increasingly home-based and prophylaxis-oriented, factors that can have tremendous patient benefits. However, the resulting potential for less frequent contact with healthcare providers limits the value of medical chart data in being able to provide a comprehensive picture of treatment outcomes and patient experiences. We designed this study to assess real-world clinical and disease burden outcomes in patients with HAE before and after use of C1INH(SC) prophylaxis using a unique hybrid design incorporating both medical chart review and qualitative, interview-based research methodologies. The combined analysis of medical chart data and patient interview narratives analyzed using qualitative methods was intended to facilitate a more holistic insight into patient outcomes in a real-world setting. We discovered that medical chart data were largely incomplete for the outcomes being studied. As a result, the analysis on attack frequency was predicated primarily on patient interview data, which were available for almost every subject and also formed the sole basis of attack severity analysis. This finding seems to provide supportive evidence for the aforementioned paradigmatic shift in HAE patient care away from acute management and high healthcare resource engagement to more independent care with less frequent need for direct interfacing with medical providers.

Across a wide range of pre-C1INH(SC) attack frequencies, there was a statistically significant and clinically meaningful reduction in HAE attack frequencies as well as in attack severity during the period after commencing LTP with C1INH(SC). Based on patient reporting, there was an 80% mean decrease in the number of attacks, which translated into approximately 31 fewer attacks PPPY on average, and medical chart data from a subgroup of patients corroborated these findings (87% mean decrease in attacks; 30 fewer attacks annually). The magnitude of attack frequency reduction observed in this real-world cohort was highly similar to the mean decrease of approximately 29 attacks PPPY experienced in both the 40 IU/kg and 60 IU/kg dose groups in the C1INH(SC) phase 3 COMPACT trial [11].

It should be noted that the mean attack frequency data in our analysis were skewed by some outlier patients, including one who self-reported an extreme attack burden that annualized to 198 yearly attacks pre-index. This patient experienced a dramatic 60% decrease in attacks post-index, but the improved rate of 78 attacks per year was still markedly higher than any other patient post-index. This patient described pre-index attack frequency as not having a break from the disease and often having only a single symptom-free day in between attack days. Given the natural course of HAE attacks, it is possible, even likely, that the patient was unable to distinguish between individual, unique attacks as opposed to persisting attack symptoms that reappeared within a couple of days despite the acute but temporary benefit of on-demand treatment. Thus, while this patient might not have actually experienced the reported number of unique HAE attacks reported, the improvement in attack-free days and overall symptom burden can clearly be interpreted. One other outlier patient reported a pre-index attack rate of 42 annual attacks and did not indicate a change in attack frequency post-index, but did report that the post-index attacks resolved more quickly. For data integrity and consistency, only patient-reported attack rates were used for the study analyses, but it should be noted that this patient’s medical chart indicated a post-index attack rate of about 1/month, or 12/year. These data highlight the clinical heterogeneity of HAE and differences in attack rates from patient to patient. Of note, the median attack frequency, which is less impacted by outlier data, decreased by 97%, from 30 attacks per year pre-index to one attack per year post-index. The patient interview data also allowed for an overall assessment of changes in attack severity. Mean severity of attacks after starting C1INH(SC) was significantly lessened by almost two-thirds (61%) relative to the preceding time period characterized by on-demand medication use only (from 2.3 to 0.9 on a scale from 0 [no/very mild attacks] to 4 [very severe]). Median attack severity decreased from moderate to mild.

Obtaining accurate data for on-demand HAE prescription use can be difficult. Typical claims database research may not capture medications obtained through specialized pharmacies. Further, given the need to pre-order on-demand medications to keep “on hand” in the event of an attack, the documented act of filling a prescription cannot be accurately interpreted as medication usage. It is also likely that family members with HAE may share on-demand medications. In our study, patient-reported rescue medication patterns and attack rates were used to estimate usage pre- and post-index, finding that mean annualized use of on-demand treatments decreased by an estimated 77% after starting C1INH(SC) prophylaxis. The median reduction in rescue medication usage, less affected by two outlier patients with particularly high attack rate burdens, was 96%. These findings track closely with reductions in rescue medication reported in the phase 3 COMPACT trial of C1INH(SC) in which mean annualized reductions in rescue medication usage were 70% and 89% with C1INH(SC) 40 IU/kg and 60 IU/kg, respectively, and corresponding median reductions were 89% and 100%, respectively [11].

HAE has been shown to have a tremendous psychological burden, even during attack-free periods. In fact, EQ-5D scores between attacks align with those reported for conditions such as asthma, chronic migraine, hemophilia, epilepsy, and multiple sclerosis [10]. Enhanced QoL has emerged as one of the predominant goals of HAE management. In the current study, two-thirds of patients reported improvements in aspects of emotional health/mental health after starting C1INH(SC), and roughly half described improvements in social life/relationships and daily activities. These findings are consistent with previously reported prospective clinical trial data [10, 12] as well as an earlier qualitative research study in patients using C1INH(SC) prophylaxis [13]. In the phase 3 COMPACT trial of C1INH(SC) and its long-term extension study, significant improvements were noted in a number of quality-of-life domains including anxiety, work productivity, and activity impairment [10, 12]. An in-depth qualitative study reported by Anderson et al. [13] provided a more granular context for quality-of-life domain improvements from the perspective of patients with HAE who experience reduced attack frequency, including factors such as no longer feeling limited by their disease, more freedom to travel, less reliance on others, healthier relationships, and improved cognition. These qualitative aspects, though difficult to measure, have undeniable personal significance and highlight the value of incorporating the use of validated patient-reported outcome measures in daily clinical practice when evaluating patients with chronic diseases like HAE.

We note a number of limitations of this study. Medical chart reviews were limited by a substantial amount of missing data, and attack frequency was the only outcome with enough data points to reasonably analyze. However, attack frequency reduction was markedly similar regardless of using chart data or interview data. Patient enrollment was by investigator invitation and required patients to have been on C1INH(SC) for ≥ 1 year, thus introducing the possibility of selection bias toward patients who had good outcomes with C1INH(SC). Consequently, the study findings may not be representative of the experience of all patients who are treated with C1INH(SC). A key objective of the study was to assess breakthrough attack rates during C1INH(SC) prophylaxis. Accordingly, at least 12 months of C1INH(SC) treatment was required to allow for the most accurate assessment of annual attack frequency, rather than extrapolating annual attack rates from a shorter time period which could confound the results, particularly in patients with an extremely low number of attacks. We note that lanadelumab became available during the data collection time window, and any patients who switched from C1INH(SC) to lanadelumab prior to 12 months of C1INH(SC) treatment would not have been eligible for the study. However, experience suggests that improved convenience, not lack of efficacy, is a major driver for switching to lanadelumab, thus we feel that the impact of excluding such patients would not have biased the outcomes of our study.

Reliance on patient-reported recall of pre- and post-index attack frequency, attack severity, and use of rescue medication is another limitation, although the statistical findings were robust and highly significant. HAE patients are typically very disease-aware and may be considered to have fairly reliable recall about frequency and severity of their attacks. Yet, patient recall bias must be recognized as a limitation of any study of this type. Ratings of attack severity were completely based on patients’ verbal descriptions during interviews, and not through the use of a quantitative scale. Quality of life was assessed qualitatively from patient interview narratives, as the largely retrospective aspect of the patient experience with C1INH(SC) precluded use of a validated questionnaire intended for measuring current or very recent QoL. The study included a relatively small cohort, although the cohort of 36 patients is relatively sizable for HAE research. There was no objective means of determining patient adherence to prescribed C1INH(SC) dosage and administration; in fact, many patients admitted to altering the dose and/or dosing frequency. However, in one sense, this adds to the value of the data in that it provides an accurate reflection of how C1INH(SC) is used in a real-world setting.

## Conclusions

Medical chart information was found to be an inadequate source of data for assessing real- world outcomes in patients with HAE using C1INH(SC) as LTP, a finding which may be evidence of the increasingly autonomous nature of HAE treatment. Qualitative patient interviews suggested that implementation of LTP with C1INH(SC) resulted in decreased HAE attack frequency and markedly lessened severity of breakthrough attacks, along with a marked decrease in rescue medication usage, all of which were of a similar magnitude to findings reported in the COMPACT phase 3 clinical study of C1INH(SC). Further, patients described improvement in multiple facets of quality of life. The study did not attempt to measure patient adherence, but the positive outcomes can be assumed to reflect a reasonable level of regimen adherence and successful dosing of a subcutaneous medication outside the oversight of a clinical trial. These are all important factors contributing to the ultimate goal of achieving total disease control and normalization of patients’ lives.

## Data Availability

The full dataset of medical chart and personal interview data gathered for this study are not publicly available. Relevant data for the purposes of this study have been de-identified for public disclosure in this manuscript.

## Abbreviations

C1INH(SC): Plasma-derived, subcutaneous C1-inhibitor
FDA: Food and Drug Administration
HAE: Hereditary angioedema
HRQoL: Health-related quality of life
IRB: Institutional review board
LTP: Long-term prophylaxis
PPPY: Per patient per year
SD: Standard deviation

## References

[1] Caballero T, Aygoren-Pursun E, Bygum A, Beusterien K, Hautamaki E, Sisic Z (2014). The humanistic burden of hereditary angioedema: results from the burden of illness study in Europe. Allergy Asthma Proc.

[2] Jindal NL, Harniman E, Prior N, Perez-Fernandez E, Caballero T, Betschel S (2017). Hereditary angioedema: health-related quality of life in Canadian patients as measured by the SF-36. Allergy Asthma Clin Immunol.

[3] Banerji A, Busse P, Christiansen SC, Li H, Lumry W, Davis-Lorton M (2015). Current state of hereditary angioedema management: a patient survey. Allergy Asthma Proc.

[4] Christiansen SC, Bygum A, Banerji A, Busse P, Li H, Lumry W (2015). Before and after, the impact of available on-demand treatment for HAE. Allergy Asthma Proc.

[5] Bygum A (2014). Hereditary angioedema—consequences of a new treatment paradigm in Denmark. Acta Derm Venereol.

[6] Bygum A, Aygören-Pürsün E, Caballero T, Beusterien K, Gholizadeh S, Musingarimi P (2012). The hereditary angioedema burden of illness study in Europe (HAEBOIS-Europe): background and methodology. BMC Dermatol.

[7] Fouche A, Saunders E, Craig T (2014). Depression and anxiety in patients with hereditary angioedema. Ann Allergy Asthma Immunol.

[8] Maurer M, Magerl M, Betschel S, Aberer W, Ansotegui IJ, Aygören-Pürsün E (2022). The international WAO/EAACI guideline for the management of hereditary angioedema—the 2021 revision and update. Allergy.

[9] Busse PJ, Christiansen SC, Riedl MA, Banerji A, Bernstein JA, Castaldo AJ (2021). US HAEA medical advisory board 2020 guidelines for the management of hereditary angioedema. J Allergy Clin Immunol Pract.

[10] Lumry WR, Craig T, Zuraw B, Longhurst H, Baker J, Li HH (2018). Health-related quality of life with subcutaneous C1-inhibitor for prevention of attacks of hereditary angioedema. J Allergy Clin Immunol Pract.

[11] Longhurst H, Cicardi M, Craig T, Bork K, Grattan C, Baker J (2017). Prevention of hereditary angioedema with a subcutaneous C1 inhibitor. N Engl J Med.

[12] Lumry WR, Zuraw B, Cicardi M, Craig T, Anderson J, Banerji A (2021). Long-term health-related quality of life in patients treated with subcutaneous C1-inhibitor replacement therapy for the prevention of hereditary angioedema attacks: findings from the COMPACT open-label extension study. Orphanet J Rare Dis.

[13] Anderson J, Levy DS, Lumry W, Koochaki P, Lanar S, Li HH (2021). Letting the patients speak: an in-depth, qualitative research-based investigation of factors relevant to health-related quality of life in real-world patients with hereditary angioedema using subcutaneous C1 inhibitor replacement therapy. Allergy Asthma Clin Immunol.

